# Nucleo-cytoplasmic shuttling of the endonuclease ankyrin repeats and LEM domain-containing protein 1 (Ankle1) is mediated by canonical nuclear export- and nuclear import signals

**DOI:** 10.1186/s12860-016-0102-z

**Published:** 2016-06-01

**Authors:** Livija Zlopasa, Andreas Brachner, Roland Foisner

**Affiliations:** Max F. Perutz Laboratories (MFPL), Department of Medical Biochemistry, Medical University of Vienna, Vienna Biocenter (VBC), Vienna, Austria

**Keywords:** Ankle1, LEM protein, Nuclease, Resolvase, Nuclear transport, Nuclear export

## Abstract

**Background:**

Ankyrin repeats and LEM domain containing protein 1 (Ankle1) belongs to the LEM protein family, whose members share a chromatin-interacting LEM motif. Unlike most other LEM proteins, Ankle1 is not an integral protein of the inner nuclear membrane but shuttles between the nucleus and the cytoplasm. It contains a GIY-YIG-type nuclease domain, but its function is unknown. The mammalian genome encodes only one other GIY-YIG domain protein, termed Slx1. Slx1 has been described as a resolvase that processes Holliday junctions during homologous recombination-mediated DNA double strand break repair. Resolvase activity is regulated in a spatial and temporal manner during the cell cycle. We hypothesized that Ankle1 may have a similar function and its nucleo-cytoplasmic shuttling may contribute to the regulation of Ankle1 activity. Hence, we aimed at identifying the domains mediating Ankle1 shuttling and investigating whether cellular localization is affected during DNA damage response.

**Results:**

Sequence analysis predicts the presence of two canonical nuclear import and export signals in Ankle1. Immunofluorescence microscopy of cells expressing wild-type and various mutated Ankle1-fusion proteins revealed a C-terminally located classical monopartite nuclear localization signal and a centrally located CRM1-dependent nuclear export signal that mediate nucleo-cytoplasmic shuttling of Ankle1. These sequences are also functional in heterologous proteins. The predominant localization of Ankle1 in the cytoplasm, however, does not change upon induction of several DNA damage response pathways throughout the cell cycle.

**Conclusions:**

We identified the domains mediating nuclear import and export of Ankle1. Ankle1’s cellular localization was not affected following DNA damage.

**Electronic supplementary material:**

The online version of this article (doi:10.1186/s12860-016-0102-z) contains supplementary material, which is available to authorized users.

## Background

The LAP2-emerin-MAN1 (LEM) protein family comprises a group of inner nuclear membrane and nucleoplasmic proteins [1, 2] with important functions in various cellular processes, including nuclear envelope architecture [3], DNA replication [4], cell cycle control [5], chromatin organization [6, 7] and the regulation of gene expression and signaling pathways [8–11]. All proteins in this family share the LEM domain, a ~40 amino acid long bi-helical motif, which binds the conserved metazoan chromatin-associated protein Barrier-to-Autointegration Factor (BAF) [12–19]. Besides the LEM domain, different additional motifs and functional domains are present in LEM proteins, such as a transmembrane domain (in the LEM proteins emerin, LEM2, MAN1, most LAP2 isoforms, and LEMD1), a carboxy-terminal winged-helix domain (present in LEM2 and MAN1), a LEM-like motif (found in all isoforms of LAP2), and ankyrin repeats in Ankyrin Repeats and LEM-domain containing proteins (Ankle) 1 and 2 [2, 14, 20–23].

Ankle1 has several unique features among the LEM protein family members. It lacks a transmembrane domain and shuttles between the cytoplasm and the nucleoplasm, and it contains a C-terminal GIY-YIG-type endonuclease domain [22, 24]. The GIY-YIG domain is the hallmark of a subgroup of the homing endonuclease superfamily [25, 26], represented mostly by group I and II introns, archaeal introns and inteins that catalyze their transfer within genomes by introducing strand breaks in intron- and intein-lacking sequences [25, 27, 28]. We have previously shown that the GIY-YIG domain of Ankle1 has nuclease catalytic activity that cuts plasmid DNA in vitro and induces DNA damage in vivo [22, 24]. Based on the findings in *C. elegans* that an inactivating mutation in the worm Ankle1 ortholog LEM3 leads to hypersensitivity of the worm mutants to various types of DNA damage, including ionizing radiation, UV-C light and DNA crosslinking agents, Ankle1/LEM3 was proposed to be involved in DNA damage repair pathways [24]. Embryos from irradiated *lem-3* mutant worms also suffer from severe defects during cell division, such as chromosome mis-segregation and anaphase bridges [24]. In mammals, however Ankle1 functions may be highly redundant, as Ankle1 knockout mice and cells are normal and did not show an impaired DNA damage response [29].

The mammalian genome contains two genes encoding proteins with a GIY-YIG nuclease domain, *Ankle1* [22] and *Slx1*, encoding Slx1 resolvase [30, 31]. Resolvases are DNA-structure specific nucleases that process and cleave Holliday junctions (HJ) [32–34]. HJs are intermediate structures of covalently linked homologous chromosomes during meiosis or of sister chromatids during homologous recombination-mediated double strand break (DSB) repair [35, 36]. HJs need to be eliminated before the end of mitosis or meiosis to assure proper chromosome segregation and to preserve genome stability. Homologous recombination followed by HJ resolution can result either in crossover (CO) events that are obligatory at a certain frequency in meiotic cells to allow exchange of genomic information, or non-crossover (NCO) events, which are preferred in mitotic cells because they avoid the loss of heterozygosity, a high risk factor for the development of cancer [37, 38]. Two major mechanisms are responsible for HJ processing in mitotic cells: the preferred dissolution by the so-called Bloom helicase BTR (BLM-TOPIIIα-RMI1-RMI2) complex that results in NCOs [39], and the resolution of HJs by structure specific endonucleases, such as the Slx4 complex (Mus81-Eme1-Slx1-Slx4) or the canonical HJ resolvase Gen1, that can lead to COs and NCOs [34, 40–42]. Because dissolution is favored over resolution in mitosis, the actions of resolvases are strictly regulated in a spatio-temporal manner [38, 43]. In human cells, Eme1, a subunit of the Slx4 complex is phosphorylated in prometaphase to stimulate interactions and activation of the Mus81-Eme1-Slx1-Slx4 complex [38]. This ensures that resolvases eliminate only HJs, which were not processed by the BTR complex in S and G2 phase. The activity of the human Gen1 resolvase is restricted to mitosis by nuclear exclusion during interphase through a leucine-rich nuclear export signal (NES) [43]. Based on the evidence of unprocessed chromatin structures in *C. elegans lem-3* mutants and the presence of a GIY-YIG nuclease domain in Ankle1, we hypothesized that Ankle1 may also function in DNA damage repair, probably redundantly with other nucleases, and its nucleo-cytoplasmic shuttling and cellular localization may be tightly controlled during the cell cycle and upon DNA damage. Hence, we wanted to identify the domains and motifs in Ankle1 involved in its nucleo-cytoplasmic shuttling and test whether DNA damage may alter Ankle1’s cellular localization. We identified one canonical nuclear localization sequence (NLS) and one nuclear export sequence (NES) in Ankle1 that mediate its translocation into and out of the nucleus, respectively. However, no changes in shuttling and/or cellular localization were found upon induction of DNA damage or during the cell cycle.

## Results

### Nucleocytoplasmic shuttling of Ankle1 is mediated by active transport mechanisms

In a previous study, we showed that Ankle1 is primarily localized in the cytoplasm in HeLa and B-cell derived RAMOS cell lines, but it accumulated in the nucleus upon inhibition of nuclear export by leptomycin [22, 44]. This suggested that Ankle1 shuttles between the cytoplasm and the nucleus. In this study, we wanted to identify the domains in Ankle1 that mediate its shuttling, and test, whether nucleo-cytoplasmic shuttling of Ankle1 and its cellular localization change upon DNA damage or during the cell cycle. Therefore we expressed Ankle1 in osteosarcoma U2OS cells, which have intact pRb and p53 pathways [45] and have frequently been used in studies investigating DNA damage repair pathways, including resolvase-mediated repair [46–48].

**Fig. 1:**
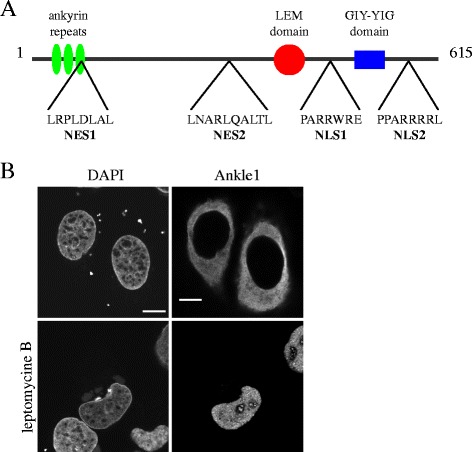
Ankle1 shuttles between nucleus and cytoplasm. **a** Schematic representation of Ankle1’s domain organization depicting predicted ankyrin repeats, the LEM domain and a GIY-YIG nuclease domain. Putative nuclear export sequences (NES1, NES2) and nuclear localization sequences (NLS1, NLS2), identified *in silico* are indicated. **b** Immuno-fluorescence analysis of ectopic Ankle1-V5 in U2OS cells without or following a 3 h treatment with 50 nM leptomycin B, an inhibitor of CRM1-mediated export. Cells were stained with antibodies to V5, and DNA with DAPI. Scale bar: 10 μm

In agreement with previous studies [22], Ankle1 ectopically expressed in U2OS cells localizes predominantly in the cytoplasm and accumulates in the nucleus upon pharmacological inhibition of the CRM1-dependent nuclear export using the drug leptomycin B (Fig. 1). I*n silico* analysis of the Ankle1 primary sequence predicted the existence of at least two nuclear localization sequences (NLS) and two nuclear export signals (NES) (Fig. 1a), suggesting that shuttling of Ankle1 may be mediated by active nuclear import and export pathways.

**Fig. 2:**
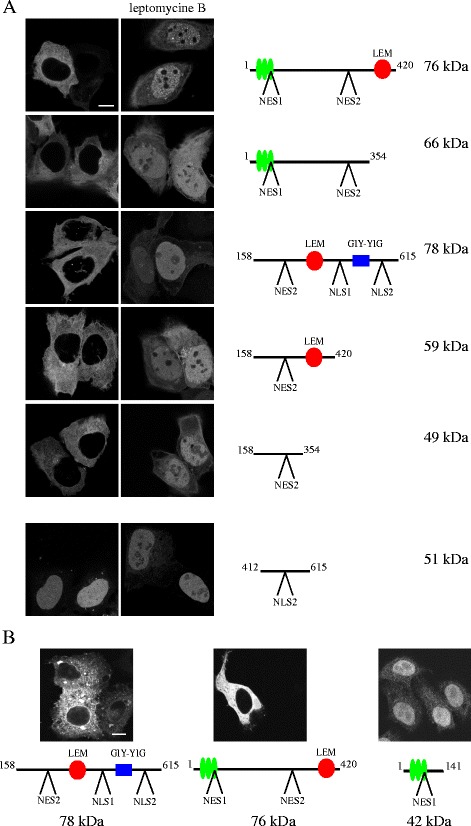
Localization of Ankle1 fragments containing different domains and export and import signals. Localization of GFP-tagged Ankle1 truncation constructs ectopically expressed in U2OS (**a**) and HeLa (**b**) cells was determined by confocal fluorescence microscopy. Molecular weights and schematic representations of domain organization of respective truncation protein constructs are indicated. Cells were fixed after 3 h of mock or leptomycin B treatment. DNA was counterstained with DAPI. Scale bar: 10 μm

In order to get a first hint whether any of the NLSs and NESs are responsible for nucleo-cytoplasmic shuttling of Ankle1, we generated a series of truncated Ankle1 constructs C-terminally fused to green fluorescence protein (GFP), and tested their subcellular localization by confocal fluorescence microscopy. Using this approach, we also wanted to test whether specific protein domains other than transport signal sequences influence Ankle1 localization. These include the ankyrin repeats, well known protein interaction domains found in many proteins [49, 50], the LEM domain, known to mediate binding to chromatin via Barrier-to-Autointegration factor (BAF) [14, 16, 51], and the GIY-YIG domain, mediating nuclease activity [25] (Fig. 1a). Ankle1^1–420^, which lacks the C-terminal domain including the GIY-YIG motif and the two NLSs, is effectively exported to the cytoplasm in both U2OS and HeLa cells (Fig. 2). Additional deletion of the LEM domain (Ankle1^1–354^) did not alter the predominant cytoplasmic localization of the construct in the absence of leptomycin B. Hence we concluded that neither the LEM domain nor the GIY-YIG domain affect nuclear export (Fig. 2). The construct lacking the N-terminal ankyrin repeats (including the predicted NES1 sequence) (Ankle1^158–615^) was efficiently excluded from the nucleus in both U2OS and HeLa cells, indicating that neither ankyrin repeats nor NES1 are essential for nuclear export and/or cytoplasmic localization. In agreement with this hypothesis, Ankle1^1–141^ containing only NES1 was not efficiently exported from the nucleus (Fig. 2b), while both Ankle1^158–420^ and Ankle1^158–354^ encompassing the central region containing only NES2 with or without the LEM domain, respectively, localize exclusively to the cytoplasm (Fig. 2a). In addition, leptomycin B-mediated inhibition of nuclear export caused efficient accumulation of most constructs in the nucleus, suggesting that a region including NES2 is sufficient for Ankle1 export. Altogether these data suggest that Ankle1 is actively exported from the nucleus predominantly through a NES2-mediated mechanism. We assume that also its nuclear import is mediated by an active NLS-mediated mechanism based on the following observations. First, Ankle1^158–615^, containing NES2 and both predicted import sequences NLS1 and NLS2 efficiently accumulates in the nucleus in the presence of leptomycin B like the wild-type protein, although its molecular weight (78 kDa) does not allow efficient nuclear translocation by passive diffusion through nuclear pores [52]. Secondly, a 51 kDa large construct containing the nuclear localization signal (NLS2) is exclusively targeted to the nucleus (Fig. 2, Ankle1^412–615^), despite its size would allow some passive diffusion out of the nucleus. We therefore conclude that Ankle1 is shuttling between the nucleus and the cytoplasm through active import and export mechanisms, but its nuclear export seems to overrule nuclear transport, as at steady-state, Ankle1 is not detected inside the nucleus.

### Mutation analyses identify NES2 and NLS2 as major transport-mediating signals

**Fig. 3:**
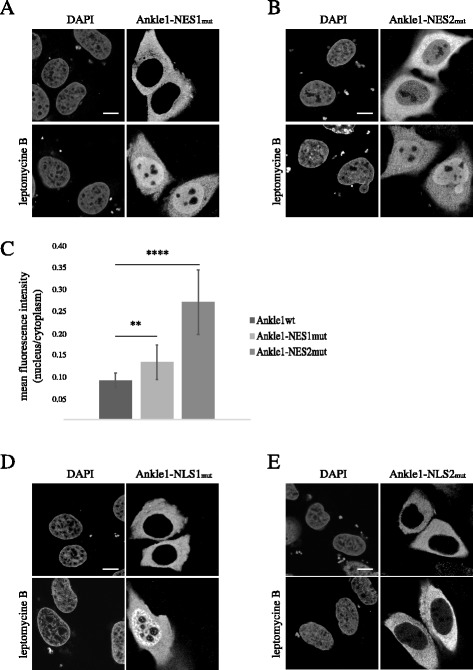
Mutation analyses identify NES2 and NLS2 as the predominant sequences controlling nucleo-cytoplasmic shuttling of Ankle1. **a**, **b**, **d**, **e** U2OS cells were transiently transfected with Ankle1-V5 carrying point mutations in NLS or NES sequences and either mock-treated or treated with leptomycin B for 3 h and processed for confocal immunofluorescence analyses using antibodies to V5 and DAPI to detect DNA. Scale bars: 10 μm. **c** Mean fluorescence intensities in nuclei and cytoplasm of cells expressing wild-type Ankle1-V5, Ankle1-NES1mut-V5 or Ankle1-NES2mut-V5 were measured in original unprocessed digital images prior to contrast/brightness adjustment and nucleus to cytoplasm signal ratios were calculated. Data were obtained from three independent experiments and analyzed using Student’s *t*-test. Ankle1-NES1, *P* = 0.002; Ankle1-NES2, *P* = 5.4E-21; *n* = 50; 15–17 cells each from three independent experiments

Our experiments using truncated Ankle1 constructs indicated that NLS2 and NES2 are the predominant regulators of active import and export, respectively. To test whether this hypothesis holds true also in the context of the full length protein, we analyzed the localization of full length Ankle1 constructs carrying point mutations at conserved residues within the nuclear transport sequences NES1, NES2, NLS1 and NLS2 (Fig. 3). Furthermore, in order to avoid potential effects of the bulky GFP tag we used the smaller V5-tag instead. Mutation of NES1 (Ankle1-NES1mut) caused only a subtle impairment of nuclear export (Fig. 3a), as indicated by the minor increase in the ratio of nucleoplasmic over cytoplasmic signal intensities of the mutant compared to wild-type control (Fig. 3c). In contrast, substitution of conserved leucine residues within the NES2 impaired nuclear export of Ankle1 and caused a significant increase in the steady-state fraction of the protein in the nucleus compared to the control (Fig. 3b, c). Given that nuclear export is predominant in defining the steady state localization of Ankle1, it was only possible to examine the consequences of NLS1 and NLS2 mutations following leptomycin B-mediated inhibition of nuclear export. Mutation of the positively charged residues within NLS1 did not affect Ankle1’s accumulation in the nucleus (Fig. 3d) under this condition, whereas mutation of NLS2 impaired nuclear import nearly completely (Fig. 3e). In summary, we concluded that NES2 and NLS2 are the predominant signals mediating active nucleo-cytoplasmic shuttling of Ankle1.

**Fig. 4:**
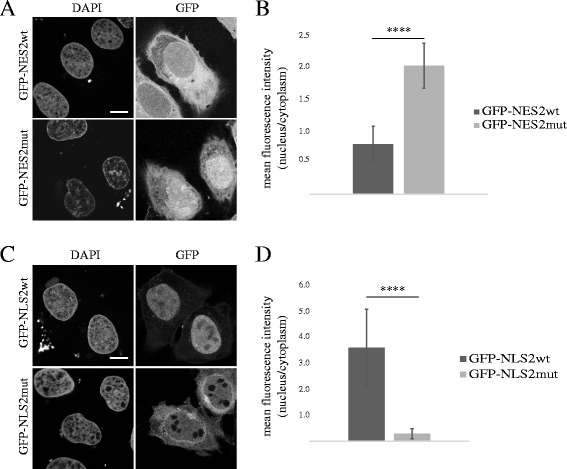
NES2 and NLS2 are functional export and import signals in heterologous reporter constructs. U2OS cells were transiently transfected with GFP constructs fused to the wild-type or mutated versions of NES2 or NLS2, fixed and prepared for fluorescence microscopy (**a**, **c**). Representative images of at least three independent experiments are shown. Scale bars: 10 μm. **b**, **d** Mean fluorescence intensities in nuclei and cytoplasm of GFP-NES2wt, GFP-NES2mut (**b**), GFP-NLS2wt or GFP-NLS2mut (**d**) transfected cells were measured in original unprocessed digital images prior to contrast/brightness adjustment, and nucleus to cytoplasm signal ratios were calculated. Data were obtained from three independent experiments and analyses were done using Student’s *t*-test. GFP-NES2mut, *P* = 7.9E-30; GFP-NLS2mut, *P* = 9.1E-18; *n* = 43; 13–15 cells each from three independent experiments

In support of this, we also show by fusing wild-type and mutated versions of NLS2 and NES2 sequences to GFP, that the wild-type, but not mutated sequences are sufficient to mediate active transport of heterologous proteins. Wild-type NES2-GFP, but not the mutated construct was efficiently excluded from the nucleus (Fig. 4a, b). Wild-type NLS2 sequence mediated efficient accumulation of the fusion construct in the nucleus, whereas the mutated version was distributed throughout the cell (Fig. 4c, d).

Overall we show that Ankle1 localization is determined predominantly by the activity of two transport signals, a nuclear export signal in the middle of the polypeptide and a C-terminal nuclear localization signal.

### Ankle1 does not change localization upon DNA damage and during mitosis

It was previously reported that a *C. elegans* mutant for *lem-3*, the worm ortholog of mammalian Ankle1, is hypersensitive to DNA damage-causing agents [24]. Furthermore, Ankle1 may be functionally related to Slx1, the only other known GIY-YIG-type endonuclease encoded in the mammalian genome, which is involved in homologous recombination-mediated repair of DNA double strand breaks in a cell cycle-regulated manner. Thus, we speculated that Ankle1 may have a similar function in DNA damage repair and set out to test whether Ankle1 may transiently accumulate in the nucleus and/or on chromatin upon induction of the DNA damage response signaling or during the cell cycle. However, as long-term expression of wild-type Ankle1 causes cell death (data not shown), we generated a stable U2OS cell line ectopically expressing an endonuclease-defective, GFP-tagged version of Ankle1 to address Ankle1 dynamics during the cell cycle and upon induction of DNA damage. The catalytically dead Ankle1 mutant shuttles between the cytoplasm and the nucleoplasm and has a predominant cytoplasmic localization at steady state like the wild-type protein (Additional file 1: Figure S1A). However, unlike for wild-type Ankle1, leptomycin B-dependent accumulation of the Ankle1 mutant in the nucleus did not induce DNA damage (Additional file 1: Figure S1B).

**Fig. 5:**
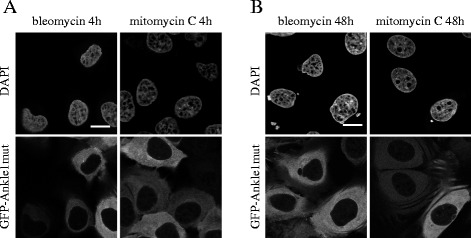
Ankle1 does not relocalize to the nucleus in response to DNA damage. U2OS cells stably expressing a catalytically inactive GFP-Ankle version were treated with the DNA damaging agents bleomycin or mitomycin C for 4 h or 48 h using high and low dosages, respectively: 3 μg/mL bleomycin, 1 μg/mL mitomycin C for 4 h (**a**), 1 μg/mL bleomycin or 0.5 μg/mL mitomycin C for 48 h (**b**). Fixed cells were imaged using confocal microscopy and representative images of at least three independent experiments are shown. Scale bars: 10 μm

To investigate potential changes in Ankle1 localization upon induced DNA damage, we treated cells with various chemical compounds, which cause different types of DNA damage and elicite different DNA repair pathways: bleomycin, a radio-mimetic drug inducing double strand breaks and mitomycin C, creating DNA crosslinks, which both are mainly repaired by non homologous end joining (NHEJ) or HR, and UV irradiation that triggers the nucleotide excision repair (NER) pathway (reviewed in [53]). Drugs were applied for short time periods at high concentration to study immediate DNA damage response (Fig. 5a) or at low dosage over a prolonged time period to cause constant DNA damage to allow activation of downstream DNA damage response signaling events (Fig. 5b). However, although the different types of treatments induced DNA damage response, as shown by the upregulation of the γH2A.X marker compared to untreated control samples (Additional file 2: Figure S2), we did not observe a (transient) change in the localization of Ankle1 nuclease-dead mutant (Fig. 5 and data not shown). Hence, Ankle1 localization may not be affected upon induction of DNA damage response signaling, or the catalytically dead mutant is unable to respond to induction of DNA damage signaling.

**Fig. 6:**
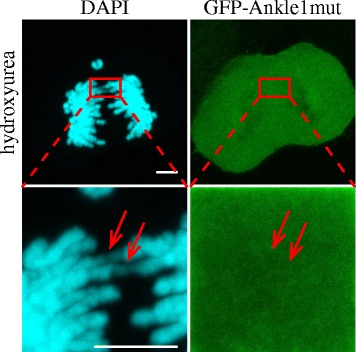
Ankle1 does not accumulate on mitotic DNA bridges induced by hydroxyurea treatment. U2OS cells stably overexpressing inactive GFP-Ankle1 were grown in the presence of 100 μM hydroxyurea for 18 h. Cells were fixed and processed for confocal fluorescence microscopy. Representative image out of at least 20 mitotic cells from three independent experiments is shown. Arrows show DAPI stained DNA bridges. Scale bar: 5 μm

Next we tested whether Ankle1, like the mammalian resolvases, may serve in DNA repair pathways during late G2 or M phase of the cell cycle, as shown for the resolvases Mus81, Slx1 and Gen1 [42, 43]. We first analyzed Ankle1 localization throughout the cell cycle and focused in particular on the potential association of Ankle1 with condensed chromosomes during mitosis. Ankle1 is uniformly distributed throughout the cytoplasm in different stages of mitosis, both in fixed (Additional file 3: Figure S3) and live cells with (Additional file 4: Movie S1) or without DNA damage induction (not shown). Unrepaired DNA damage and persistent HJs were shown to lead to defects in chromosomal segregation during anaphase [47], visible as small DNA fibers connecting separated chromatids (so-called anaphase bridges). Resolvases are known to be activated upon such mitotic defects and to localize to these anaphase bridges [54] in order to resolve HJs and to allow mitotic progression without chromosomal mis-segregation. We tested whether Ankle1 shows a similar re-localization to anaphase bridges following treatment of cells with the replication inhibitor hydroxyurea to increase the frequency of such mitotic defects. Screening mitotic figures in three independent experiments did not reveal re-localization and/or accumulation of Ankle1-dead mutants to anaphase bridges under the given experimental setup (Fig. 6, arrows).

## Discussion

In this study we show that Ankle1 endonuclease shuttles between the cytoplasm and the nucleus by an active NLS-mediated nuclear import and a NES-mediated nuclear export, despite its predominant steady-state localization in the cytoplasm. Using various Ankle1 deletion constructs and full length Ankle1 with mutated, non-functional transport signals, we find that nucleo-cytoplasmic shuttling is predominantly achieved by the concerted actions of a C-terminal monopartite NLS sequence [55] and a canonical *rev*-type NES sequence [56] in the central region of Ankle1 polypeptide. As Ankle1 efficiently accumulates in the nucleus upon treatment with leptomycin B, a specific inhibitor of CRM1 [57], Ankle1 is likely excluded from the nucleoplasm via a CRM1-dependent nuclear export. In silico analysis predicted two potential nuclear export sequences fitting the highly conserved leucine-rich NES sequence motif (LxxxLxxLxL, reviewed in [58]), one located within the ankyrin repeats of Ankle1, the other in the central region between the ankyrin repeats and the LEM domain. Experimental testing showed that only the latter was sufficient to mediate efficient nuclear export of tested Ankle1 constructs (Figs. 2 and 4a, b). Similarly, among the two predicted canonical monopartite NLSs present in Ankle1 polypeptide, NLS2 at the very C-terminus was found to mediate nuclear import. Several observations speak in favor of an active NLS- and NES-mediated transport of Ankle1, rather than a so-called “piggy-back” mechanism in which Ankle1 is co-transported with other proteins: First, wild-type Ankle1 but not Ankle1 mutants with a mutated nonfunctional NES2 sequence were exported from the nucleus (Fig. 3b). Second, Ankle1 mutants with a mutated nonfunctional NLS2 sequence, unlike wild-type protein, were not imported into the nucleus following leptomycin B treatment (Fig. 3e). Third, both NLS2 and NES2 sequences were able to mediate effective import and export, respectively of a heterologous protein (Fig. 4).

Despite the presence of an active NLS sequence, Ankle1’s steady state localization is predominantly cytoplasmic. In fact we were unable to detect any signal above background in the nucleus by fluorescence microscopy. This observation raises the question, whether additional factors may be involved favoring nuclear export over import or whether the predominant cytoplasmic localization is merely a consequence of a tightly regulated balance between import and export rates. Potential additional mechanisms involved in the regulation of Ankle1 nucleo-cytoplasmic shuttling may involve posttranslational modifications of Ankle1 or specific retention in the cytoplasm by interaction with cytoskeletal components, but currently there is no evidence for any of these pathways being involved.

What may be the physiological relevance of nucleo-cytoplasmic shuttling of Ankle1? Constant shuttling of Ankle1 across the nuclear envelope, while primarily localizing in the cytoplasm may prevent accidental damage in the genome caused by Ankle1’s endonuclease activity. This hypothesis is supported by previous findings showing that leptomycin B-mediated accumulation of Ankle1 in the nucleus causes DNA cleavage and cell death [22]. This hypothesis also predicts that the localization of Ankle1 has to be regulated in a tightly controlled manner dependent on DNA damage and/or the cell cycle as shown for other nucleases:

DNAseI, a nuclease involved in apoptosis, is excluded from the nucleus by association with cytoplasmic actin, and association is stabilized by cofilin and disrupted by *N*-gelsolin [59].

A tight regulation of nucleases was also reported for HJ-processing resolvases, including Slx1, the only other GIY-YIG domain-containing protein besides Ankle1 in the human genome [30, 31]. Dissolution of HJs by the BTR complex is the preferred mechanism in mitotic cells, because it avoids CO formation [39], but HJs, which could not be processed or escaped the BTR complex-mediated repair can be resolved by one of the three structure specific endonucleases: Slx1-Slx4, Mus81-Eme1, or Gen1. In order to allow the preferred processing of HJs by BTR and use resolvases only as a backup mechanism in G2 and M-phase of the cell cycle, resolvase activity is tightly regulated throughout the cell cycle. Slx1 is active only in a complex with Slx4. Slx1 forms homodimers in G1 and S-phase of the cell cycle [60], and formation of the active Slx4 complex (Mus81-Eme1-Slx1-Slx4) is only promoted in prometaphase by phosphorylation of Eme1 by cyclin dependent kinases (CDKs) [38]. Gen1 is regulated at the level of nuclear exclusion via a leucine-rich NES sequence [43], assuring that only breakdown of the nuclear envelope during mitosis allows Gen1 to access unresolved DNA bridges.

Based on these recently reported findings on the regulation of resolvases we tested whether Ankle1 localization may be changed transiently upon inducing DNA damage or during the cell cycle. However neither long-term treatment at low doses nor short treatments with higher doses of mitomycin C, UV-C and bleomycin changed Ankle1 localization. Similarly, Ankle1 did not change its localization during the cell cycle and did not associate with anaphase bridges [47, 54] in mitotic cells treated with hydroxyurea. One caveat of these studies is that we had to use a catalytically inactive Ankle1 mutant containing mutations in its GIY-YIG domain, as the wild-type protein causes cell death and precluded cell cycle dependent analyses. As the catalytically dead mutant showed the same nucleo-cytoplasmic shuttling and steady state cytoplasmic localization, we consider it unlikely that the mutation in the GIY-YIG motive affects its cellular localization. Therefore, we concluded that Ankle1 localization is neither affected by activated DNA damage response signaling nor cell cycle stages in U2OS cells. Alternatively the high redundancy of HJ processing pathways may obstruct the analysis of specific Ankle1 functions in HJ processing under these conditions.

Although data obtained in *C. elegans* [24] and recent reports on a potential linkage of single nucleotide polymorphisms in the human Ankle1 gene with an increased risk for certain cancers [61, 62] are consistent with a function of Ankle1 in DNA damage response pathways, we cannot exclude that it is involved in other cellular processes. In this study, we elucidated one level in the regulation of Ankle1 localization by identifying active NLS and NES sequences.

## Conclusions

This study identifies a centrally located *rev*-type CRM1-dependent NES in the Ankle1 polypeptide and a C-terminal canonical mono-partite NLS, which together mediate shuttling of Ankle1 between the cytoplasm and the nucleus and maintain a predominantly cytoplasmic localization at steady state. Induction of DNA damage response signaling did not affect cellular localization or chromatin association of Ankle1 leaving it unclear, if and how Ankle1 localization may be regulated upon induction of DNA damage response signaling.

## Methods

### Cell culture and transfection

U2OS and HeLa cells were cultivated in Dulbecco’s modified Eagle’s medium (DMEM, PAA, Pasching, Austria) supplemented with 10 % fetal calf serum (Invitrogen, Carlsbad, CA), 100 U/mL penicillin, 100 μg/mL streptomycin and 2 mM L-glutamine at 37 °C and 8.5 % CO_2_. Transient transfections were carried out using the Nanofectin kit as stated in the manufacturer’s instructions (PAA). Inhibition of CRM1-dependent nuclear export was performed using 10 ng/mL leptomycin B (Enzo Life Sciences, Lausen, Switzerland) for three hours. A stable U2OS cell line expressing catalytically dead Ankle1 mutant was generated by transfection of peGFP-Ankle1mut followed by antibiotic selection (200 μg/mL G418) and single cell clone expansion.

### Plasmids and cloning

Site directed PCR mutagenesis was performed following the manufacturer’s protocol (Stratagene, Santa Clara, CA) by amplification of pEntry-Ankle1 [22] using primers containing the desired point mutations and verified by sequencing. Primers are listed in Table 1. Subsequently, the obtained Ankle1 mutants were shuttled via Gateway cloning (Invitrogen) into a Gateway-compatible pTracerB plasmid [22].

**Table 1:** Primer sequences used for site directed PCR mutagenesis and molecular cloning

NES1mut1-F	CCGGCGGACGCGGCCGCGCAGCAGGGACACCTGGA
NES1mut1-R	TGCTGCGCGGCCGCGTCCGCCGGCCGGAGTCCGTC
NES1mut2-F	CGCGACCAGGACGGAGCTCGGCCGGCGGAC
NES1mut2-R	GCCGCGTCCGCCGGCCGAGCTCCGTCCTGG
NES2mut1-F	TGCAGGCCGCGACTGCGACCCCACCAAATG
NES2mut1-R	GGTGGGGTCGCAGTCGCGGCCTGCAGACGC
NES2mut2-F	AGAAGCGAATGCCCGTGCGCAGGCCCTGAC
NES2mut2-R	GCCTGCGCACGGGCATTCGCTTCTGCCTCC
YIGmut-F	ACTTTCATCCGTGCCATCTTCGCCGCGGCCAAAGG
YIGmut-R	CCTTTGGCCGCGGCGAAGATGGCACGGATGAAAGT
GFP-NES2-F	AATGAATTCGCCCTGGGCTGGGTCATTG
GFP-NES2-R	CCGGTCGACTTACAGGAGAGGCATGGAGGAAGG
GFP-NLS2-F	AGGGAATTCGGAGGCGTGTATTGTGGAAG
GFP-NLS2-R	AGTGTCGACTTACTTCAGCCAGGAAGACAAGG
Ankle1^1^	AGCGTCGACATGTGCTCGGAGGCCCGCCTGG
Ankle1^141^	GTTCTAGACGATCCGGGTCCGGGTCCTG
Ankle1^158^	CACCGTCGACATGTCTGGACCTACCGATGAGAC
Ankle1^354^	CATTCTAGACCGGCAAGGGCCGACAG
Ankle1^412^	CACCGTCGACATGGCCCTGCGGACGGGCTGTATTC
Ankle1^420^	TATAAGCTTTGGAATACAGCCCGTCCGCAGG
Ankle1^615^	GTATCTAGAGCCCCGGGCCTGGATGTC

GFP-tagged Ankle1 truncation constructs were generated by amplifying the DNA sequence encoding amino acids 1–141, 158–615, 158–420, 158–354, 1–354, 1–420 and 412–615 by PCR. The oligos used for PCR (see Table 1) contained a SalI restriction site within the forward and a XbaI restriction site in the reverse primer, the generated PCR products were cut and ligated into the SalI/XbaI sites of peGFP-C1 (Clonetech, Mountain View, CA).

GFP-NES2wt and GFP-NES2mut fusion constructs were created by the PCR amplification of pEntry-Ankle1-NES2wt or pEntry-Ankle1-NES2mut (amino acids 246–319) (for primers see Table 1) and cloned into peGFP-C1 vector using EcoRI and SalI restriction sites. GFP-NLS2wt and GFP-NLS2mut were created using the same restriction sites after amplification of the fragment (amino acids 561–603) from the respective pEntry-Ankle1-NLS2wt or pEntry-Ankle1-NLS2mut plasmids.

Catalytically dead GFP-Ankle1mut was generated by PCR-based introduction of point mutations within the GIY-YIG motif, replacing GIY by AAA using primers as shown in Table 1.

### Immunofluorescence microscopy and image analysis

Cells were grown on glass coverslips or seeded onto Ibidi-treat microscopy slides for live-cell imaging. Cells on coverslips were washed twice with PBS, fixed in PBS containing 4 % paraformaldehyde for 10 min and permeabilized with 0.5 % Triton-X-100 in PBS for 5 min. Fixed cells were rinsed three times with PBS and blocked with 3 % BSA in PBS for at least 1 h. Cells transfected with Ankle1-V5 constructs were incubated with a mouse anti-V5 primary antibody (Invitrogen) diluted 1:200 in blocking solution for 45 min at room temperature, washed three times with PBS and probed with a fluorescently labeled secondary antibody (DyLight 650, Thermo Scientific, Waltham, MA) as described for the primary antibody. All samples were counterstained with 100 ng/mL DAPI (Sigma, Munich, Germany) for 5 min and mounted in Mowiol (Fluka, Buchs, Switzerland).

Images were acquired on a confocal laser scanning microscope (LSM 710, Carl Zeiss, Jena, Germany) using a 63x/1.4 NA Plan-Apochromat oil immersion objective. Live-cell imaging was performed on a spinning disc microscope, using a 63x/1.4 NA Plan Apochromat DIC oil immersion objective (Visitron Systems, Germany).

Mean fluorescence intensities were measured and quantified in ImageJ (NIH, Bethesda, MD) using a short script (“macro”) that creates a mask of the nucleus based on the DAPI stain and measures fluorescence intensity within this area in the V5 or GFP channel. Fluorescence intensity in the cytoplasm was calculated by scanning the mask of the whole cell (as stained in the V5 or GFP channel), from which we subtracted fluorescence intensity measured in the nucleus. Quantification of fluorescence intensities was done using the original, unprocessed raw data (without contrast and brightness adjustments). Digital images for production of printable figures were adjusted for brightness and contrast and exported using the LSM Image-Browser (Zeiss) and Adobe Illustrator (Adobe Systems, San Jose, CA).

## Abbreviations

CO, crossover; DSB, double strand break; GFP, green fluorescence protein; HJ, Holliday Junction; HR, homologous recombination; LEM, LAP-Emerin-MAN; NCO, non-crossover; NES, nuclear export signal; NLS, nuclear localization signal

## Additional files

Additional file 1: Figure S1. Catalytically dead Ankle1 mutant shuttles like wild-type Ankle1 but does not induce DNA damage response. (**A**) U2OS cells stably expressing GFP-Ankle1mut (GIY-YIG to GIY-AAA) were imaged using confocal microscopy without or after 3 h leptomycin B treatment. (**B**) U2OS cells were transiently transfected with Ankle1wt-V5 or Ankle1mut-V5 (GIY-YIG to GIY-AAA). Cells were analyzed by confocal immunofluorescence microscopy following staining with antibodies to V5 and γH2A.X. Scale bars: 10 μm. (PDF 990 kb) Additional file 2: Figure S2. DNA damage response is active in U2OS cells stably overexpressing GFP-Ankle1mut. Cells were fixed and stained for γH2A.X either untreated or after overnight treatment with bleomycin (1 μg/mL), mitomycin C (0.5 μg/mL) or hydroxyurea (100 μM) and imaged using confocal microscopy. Scale bar: 10 μm. (PDF 976 kb) Additional file 3: Figure S3. Ankle1 does not accumulate on condensed chromosomes during mitosis. U2OS cells stably expressing GFP-Ankle1mut were fixed, stained with DAPI and imaged using a LSM700 confocal microscope. Representative images of at least five cells per mitotic phase from three independent experiments are shown. Scale bar: 10 μm. (PDF 1788 kb) Additional file 4: Movie S1. Ankle1 does not relocalize from the cytoplasm upon treatment with mitomycin C. Living U2OS cells expressing GFP-Ankle1mut were imaged for 22 h (upper left corner) in the presence of 1 μg/ml mitomycin C on a spinning disc microscope. Time interval between images is 7 min. (AVI 340222 kb)
