# Supplementary figures and images for: Nucleo-cytoplasmic shuttling of the endonuclease ankyrin repeats and LEM domain-containing protein 1 (Ankle1) is mediated by canonical nuclear export- and nuclear import signals
Source: BMC Cell Biol. 2016 Jun 1;17:23. doi: 10.1186/s12860-016-0102-z (PMC4888674; doi:10.1186/s12860-016-0102-z)

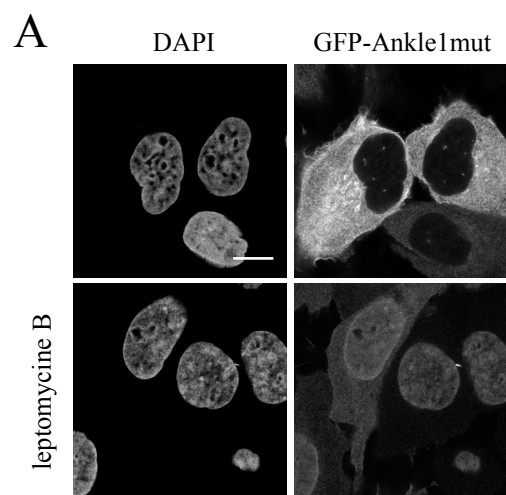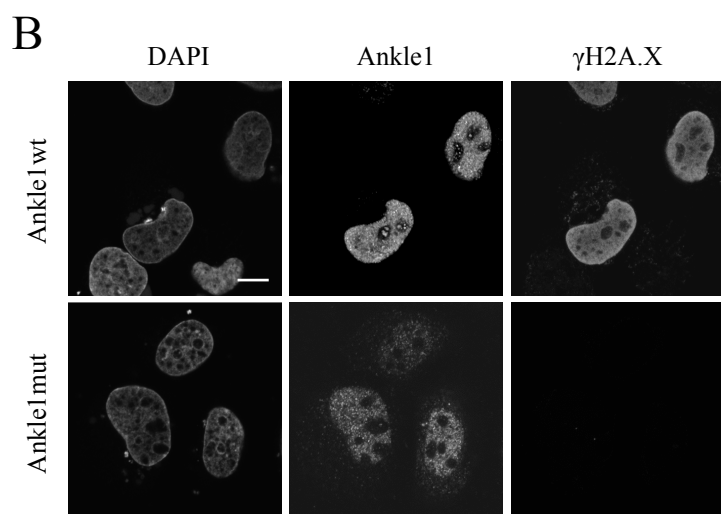

Supplement: Additional file 1: Figure S1. — Catalytically dead Ankle1 mutant shuttles like wild-type Ankle1 but does not induce DNA damage response. (A) U2OS cells stably expressing GFP-Ankle1mut (GIY-YIG to GIY-AAA) were imaged using confocal microscopy without or after 3 h leptomycin B treatment. (B) U2OS cells were transiently transfected with Ankle1wt-V5 or Ankle1mut-V5 (GIY-YIG to GIY-AAA). Cells were analyzed by confocal immunofluorescence microscopy following staining with antibodies to V5 and γH2A.X. Scale bars: 10 μm. (PDF 990 kb) [file 12860_2016_102_MOESM1_ESM.pdf]

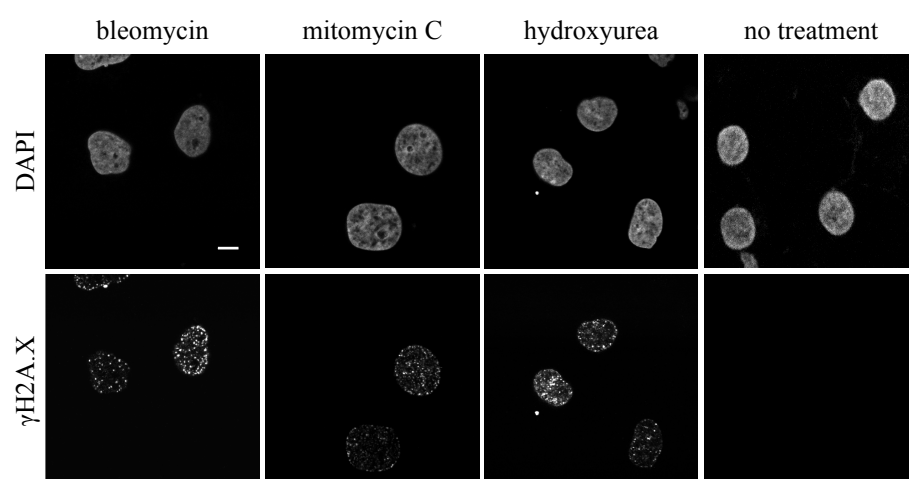

Supplement: Additional file 2: Figure S2. — DNA damage response is active in U2OS cells stably overexpressing GFP-Ankle1mut. Cells were fixed and stained for γH2A.X either untreated or after overnight treatment with bleomycin (1 μg/mL), mitomycin C (0.5 μg/mL) or hydroxyurea (100 μM) and imaged using confocal microscopy. Scale bar: 10 μm. (PDF 976 kb) [file 12860_2016_102_MOESM2_ESM.pdf]

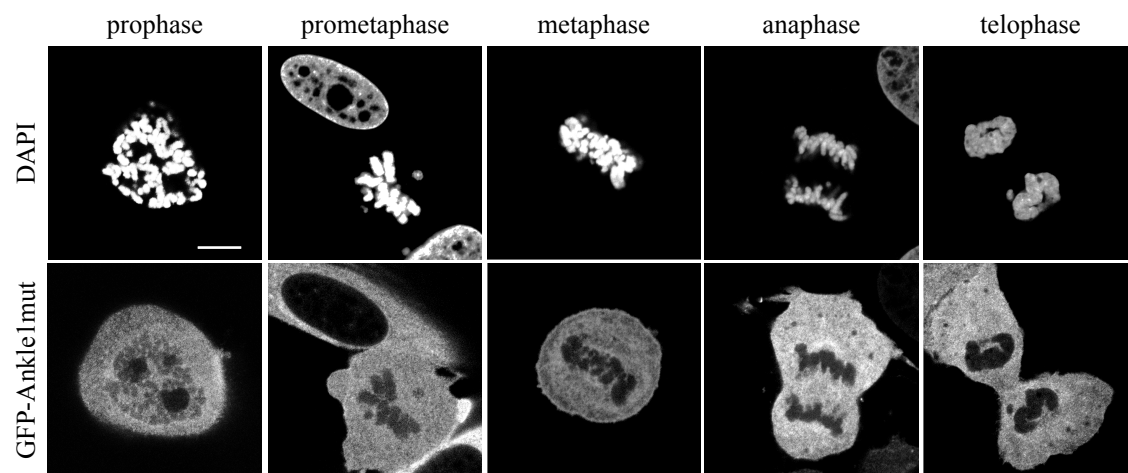

Supplement: Additional file 3: Figure S3. — Ankle1 does not accumulate on condensed chromosomes during mitosis. U2OS cells stably expressing GFP-Ankle1mut were fixed, stained with DAPI and imaged using a LSM700 confocal microscope. Representative images of at least five cells per mitotic phase from three independent experiments are shown. Scale bar: 10 μm. (PDF 1788 kb) [file 12860_2016_102_MOESM3_ESM.pdf]
